# Morphological differences between habitats are associated with physiological and behavioural trade-offs in stickleback (*Gasterosteus aculeatus*)

**DOI:** 10.1098/rsos.160316

**Published:** 2016-06-29

**Authors:** Frank Seebacher, Mike M. Webster, Rob S. James, Jason Tallis, Ashley J. W. Ward

**Affiliations:** 1 School of Life and Environmental Sciences A08, University of Sydney, New South Wales 2006, Australia; 2 School of Biology, University of St Andrews, St Andrews, UK; 3 Centre for Applied Biological and Exercise Sciences, Coventry University, Coventry CV1 5FB, UK

**Keywords:** population, locomotor performance, salinity, social behaviour, muscle

## Abstract

Local specialization can be advantageous for individuals and may increase the resilience of the species to environmental change. However, there may be trade-offs between morphological responses and physiological performance and behaviour. Our aim was to test whether habitat-specific morphology of stickleback (*Gasterosteus aculeatus*) interacts with physiological performance and behaviour at different salinities. We rejected the hypothesis that deeper body shape of fish from habitats with high predation pressure led to decreases in locomotor performance. However, there was a trade-off between deeper body shape and muscle quality. Muscle of deeper-bodied fish produced less force than that of shallow-bodied saltmarsh fish. Nonetheless, saltmarsh fish had lower swimming performance, presumably because of lower muscle mass overall coupled with smaller caudal peduncles and larger heads. Saltmarsh fish performed better in saline water (20 ppt) relative to freshwater and relative to fish from freshwater habitats. However, exposure to salinity affected shoaling behaviour of fish from all habitats and shoals moved faster and closer together compared with freshwater. We show that habitat modification can alter phenotypes of native species, but local morphological specialization is associated with trade-offs that may reduce its benefits.

## 1. Introduction

Specialization to different environmental conditions provides fitness advantages within local habitats [1–3]. Hence, environmentally sensitive production of different phenotypes can increase the resilience of species to environmental change. Specialization may result from differential selection pressures within habitats [2], which may ultimately lead to genetic structuring of populations and reproductive isolation between subpopulations [4]. For example, stickleback (*Gasterosteus aculeatus*) diverged genetically in response to different environmental salinities [5,6] and in response to different predation pressures [7]. Alternatively, environmental conditions experienced by parents and grandparents as well as during early post-zygotic development can alter gene expression programmes and result in phenotypes that are specialized to local habitats without affecting DNA sequences [8]. For example, environmental temperature and salinity can affect morphology and growth in stickleback via their effect of developmental processes [9–11].

Minor differences in morphology can be associated with pronounced differences in resource acquisition and predator escape [12–14]. For example, deeper body shape increases escape from gape-limited fish predators [15]. Morphological changes, however, can have consequences beyond affecting resource acquisition and predator–prey interactions. For example, deeper body shape can also influence locomotion of animals, particularly in water where deeper bodies increase hydrodynamic drag [16]. As a consequence, there may be a trade-off between responses to predators and locomotor performance. Locomotor performance is ecologically important because it facilitates foraging and dispersal and it is related to reproductive fitness [17–19]. Locomotor performance also has a strong effect on behaviour [20,21]. In group-living animals, such as many fish species including stickleback, the cohesion of the group is particularly important to maintain the fitness benefits that are afforded by social behaviour [22–24]. Any changes in locomotor performance may alter the integrity of a moving group and therefore reduce the benefits of grouping behaviour. Hence, individuals would have an advantage if morphology-induced reductions in locomotor performance could be compensated, for example by muscle with greater force production [25–27] to overcome increased drag.

These relationships are further complicated if there are also gradients in abiotic factors such as temperature or salinity. In aquatic coastal environments, there are often salinity gradients that can have pronounced effects on fish physiology and morphology [28,29]. For example, increased salinity led to decreased body size, lower juvenile survival rates and worse body condition in stickleback [30]. Exposure to salt water also led to shallower bodies in stickleback [11], which may improve swimming performance by reducing drag [31,32], although shallower bodies may also be associated with reduced muscle mass which can have a negative effect on swimming [33]. Salinity can also reduce chemical communication between fish and thereby alter shoaling behaviour [34]. The effects of salinity on the physiology underlying locomotor performance can therefore interact with intra-specific communication to determine the function of shoals. However, fish can compensate at least partially for the effects of salinity. Fish from populations experiencing chronic high salinities performed better when exposed to high salinity than those originating from freshwater populations [30], indicating that genetic adaptation or developmental modifiers match phenotypes to their prevailing conditions [35]. Our aim was to determine whether morphological changes of stickleback in response to different habitats within a heterogeneous environment led to divergence in locomotor performance and behaviour.

In an earlier study [36], we have shown that there are significant differences in the morphology of stickleback from river, saltmarsh and ditch habitats within the same drainage system in eastern England. We now sampled the same habitats within that drainage system to determine whether fish from different habitats were still different morphologically. The habitats we selected differed in predation pressure and abiotic characteristics [36]. The river habitat contains relatively high densities of piscivorous fish predators, such as Eurasian perch (*Perca fluviatilis*) and northern pike (*Esox lucius*). By contrast, ditch and saltmarsh environments contained no fish predators. Salinity was greatest in the saltmarsh (20 ppt) followed by the ditch (10 ppt) and river (0 ppt) habitats. Temperature was similar in all habitats. We tested the hypothesis that deeper body shape leads to reductions in swimming performance. An alternative is that fish with deeper bodies have greater muscle endurance and force production to overcome increased drag. Salinity gradients may cause a trade-off so that fish optimize osmoregulatory responses at their habitat salinity at the cost of decreased performance if conditions diverge [37]. Hence, we tested the hypothesis that fish from different populations perform best at the salinity predominant at their habitat. Alternatively, animals may be able compensate for abiotic changes in their environment by developmental or reversible acclimation so that salinity has no effect on swimming or muscle performance.

## 2. Material and methods

### 2.1. Study animals and study sites

Stickleback (*G. aculeatus*) were collected using dipnets from six sites in the Great Eau drainage in Lincolnshire (within 10 km of 53.37° N, 0.18° E), England during July 2015, which is the same area where we sampled fish in 2005 [36]. We sampled two sites each within three distinct habitat types: river channels (site 1: 53°22'11.13′′ N, 0°.11'21.85′′ E, site 2: 53°16'54.51′′ N, 0°.13'35.11′′ E; salinity = 0 ppt, temperature = 18.4°C), a network of man-made drainage ditches (site 1: 53°26'00.59′′ N, 0°.10'45.37′′ E, site 2: 53°25'44.80′′ N, 0°.11'07.29′′ E; salinity = 10 ppt, temperature = 18.7°C), and a coastal saltmarsh system (site 1: 53°25'59.87′′ N, 0°.10'49.11′′ E, site 2: 53°25'48.92′′ N, 0°.11'04.73′′ E; temperature = 18.7°C). Note that the two saltmarsh sites were distinct pools that were connected only during spring tides and floods. The ditch and saltmarsh sites were separated by a levee and there was no flow between them. Sticklebacks are common in all habitat types and all fish were fully plated. The saltmarsh fish were taken from ponds separated by a 150 m stretch of land. The ditch fish were separated by a 0.8 km stretch of water, the majority of which contained extremely dense vegetation including grass and reeds. The two river sites were separated by 8 km. We measured water temperatures at the capture sites with a digital thermometer (Traceable Digital Thermometer, Control Company, Friendswood, TX, USA) and salinity with a refractometer (Red Sea, Houston, TX, USA).

In the laboratory, we maintained fish at 18°C in water from their capture sites in round plastic containers (0.9 m diameter, 0.25 m water depth), separating fish from different habitats and sites. Water temperature was maintained with a water chiller (HC150A, Hailea Co. Ltd, China). Fish were held at either 0 or 20 ppt salinity for subsequent experiments in freshwater or at 20 ppt, respectively. Tanks were continuously aerated. We did not re-use individual fish for different experimental measures. Fish were kept in captivity for 12–18 h before measurements were taken and we released fish at their capture sites after experiments, except for muscle mechanics experiments. We did not feed fish.

### 2.2. Morphometric measurements

**Figure 1.**
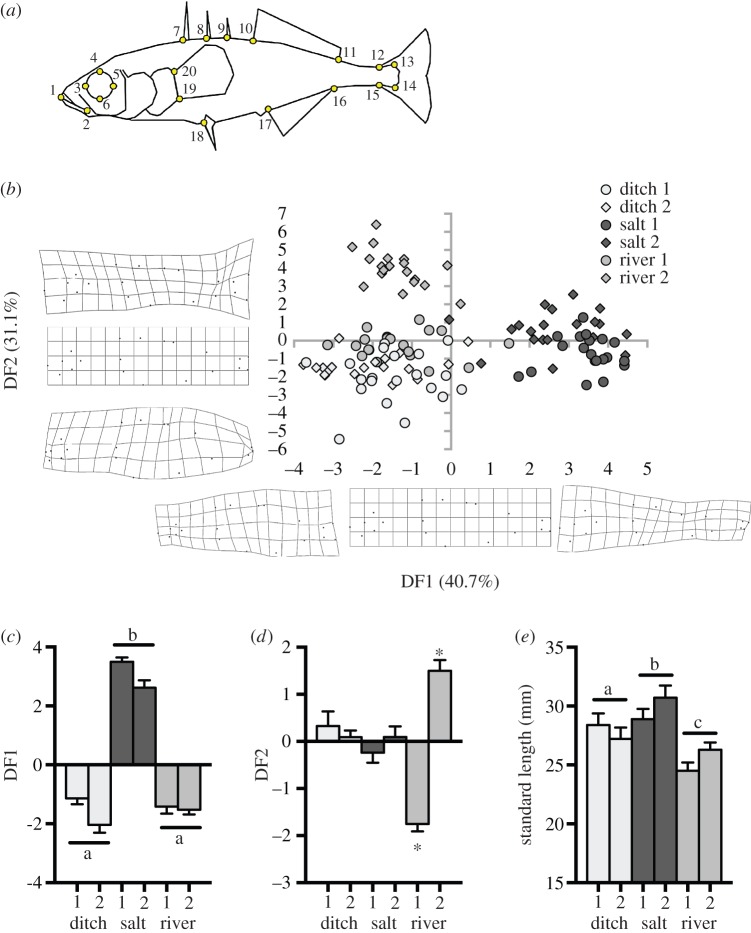
Morphometric characteristics of stickleback from different habitats. We determined the dominant discriminant functions (DF1 and DF2) from an analysis of Landmark distances (*a*) to test whether morphology differed between fish (*n* = 19–20 per site) from the two sites within each of the ditch (ditch 1 and ditch 2), saltmarsh (salt 1 and 2) and river (river 1 and 2) habitats (*b*). There were significant differences in DF1 and DF2 between habitats (*c*,*d*), but sites within habitats differed only with respect to DF2 within the river habitat (*d*). Note that absolute values for DF1 and DF2 are shown in (*b*), but (*c*) and (*d*) show length-corrected residuals. Note also that the deformation plots shown in (*b*) show exaggerated (×3) differences in body shape, for easier visualization. Standard length differed significantly between sites (*e*). Means ± s.e. are shown. Horizontal bars with different letters indicate differences between groups of bars and asterisks indicate differences between bars within groups.

We performed image landmarking and morphological analysis to quantify variation in the body shape collected from the different sites. This was performed for 20 fish from each of the two saltmarsh sizes, the first ditch and the first river site, and 19 fish each from the second ditch and river sites, for a total sample size of 118 fish. Each individual fish was laid on its right flank within a groove in a polystyrene sheet, to prevent deformation of the body along its length, and a digital photograph of the left flank was taken using a digital camera (Lumix FZ200, Panasonic, Japan). The polystyrene block was secured to the bench with tape and the camera tripod was set in the same position for all samples. From the photographs, we used the program TpsDIG [38] to record the *x* and *y* coordinates of 20 landmarks from each fish. Landmark locations are shown in figure 1*a*. Prior to collecting the landmarks, the images were shuffled, in order to guard against any unconscious systematic bias in landmark placement. The coordinate data obtained using TpsDIG was processed using the program TpsRelw [38], which uses principal component analysis to derive a set of partial warp and uniform score values describing shape variation within the sample. Discriminant function analysis (DFA) was then used to place these partial warp and uniform score values into discriminant functions. Finally, TpsRegr [38] was used to obtain visualization plots of body shape variation within the sample. This program performs a regression between the coordinates captured by TpsDIG and the discriminant variables obtained from the DFA to produce grid deformation plots (figure 1*b*).

### 2.3. Swimming performance

We measured critical sustained swimming speed (*U*_crit_), which is a standard measure of fish swimming performance. We measured *U*_crit_ in a clear plastic swimming flume (150 mm length × 32 mm diameter), which fitted tightly over the intake end of a submersible inline pump (12 V DC, iL500, Rule, Hertfordshire, UK). The pump drew water through the flume and the flume was separated from the pump by a plastic grid. A bundle of hollow straws at the inlet of the flume helped maintain laminar flow. The flume was contained in a plastic tank (645 × 423 × 276 mm). We used a variable power source (NP9615; Manson Engineering Industrial, Hong Kong, China) to adjust the flow speed by altering the DC voltage delivery to the pump. A flow meter (6710 M, DigiFlow, Savant Electronics, Taichung, Taiwan) was connected to the pump to provide flow rate in real time. The swimming protocol followed by Dalziel & Schulte [39], and fish were swum initially for 10 min at 3 cm s^−1^, then flow velocity in the flume was increased in steps (*U*_i_) of 3 cm s^−1^ every 5 min (*T*_i_). *U*_crit_ was determined as *U*_crit_ = *U*_f_ + *T*_f_/*T*_i_ × *U*_i_, where *U*_f_ is the highest speed maintained for an entire interval and *T*_f_ is the time until exhaustion at the final speed interval. Fish were considered to be exhausted when they could no longer keep their position in the water column after two chances; that is, when the fish first fell back on the plastic grid, water flow was reduced immediately until the fish swam again and then increased again to the previous velocity. The next time the fish fell back, the trial was ended.

*U*_crit_ was measured in 12 fish from each site within each habitat and in each fresh and saline (20 ppt) water (i.e. in a total of 2 sites × 3 habitats × 2 salinities × 12 fish = 144 fish). We took a photograph of each fish and determined standard length from the digital image in GraphClick software (v. 3.0.2, Arizona Software, USA). Salinity was increased in the saline treatment by adding aquarium salt to the water and salinity was measured with a refractometer (Red Sea, Houston, TX, USA).

### 2.4. Muscle biomechanics

Fish used to determine biomechanics of isolated muscle were euthanized via a blow to the head and the spinal cord was transected. The skin was removed and a section of rostral muscle fibres of 7 to 8 myotomes in length was dissected from one side of the fish for measurements of muscle mechanics. Dissections were conducted in cooled (less than 5°C) aerated fish Ringer solution (composition in millimole per litre: NaCl 115.7; sodium pyruvate 8.4; KCl 2.7; MgCl_2_ 1.2; NaHCO_3_ 5.6; NaH_2_PO_4_ 0.64; HEPES sodium salt 3.2; HEPES 0.97; CaCl_2_ 2.1; pH 7.4 at 20°C). The spine was removed from the muscle preparation, except that we left one myotome attached to the residual amount of spine at either end of the preparation. We clamped the remaining spine with crocodile clips attached to a strain gauge at one end (UF1, Pioden Controls Ltd, Canterbury, Kent, UK), and at the other end to a motor arm (V201, Ling Dynamics Systems, Royston, Hertfordshire, UK) attached to a linear variable displacement transformer (LVDT; DFG 5.0, Solartron Metrology, Bognor Regis, Sussex, UK). Each muscle preparation was then allowed to equilibrate for 10 min at 18.0 ± 0.5°C in circulating aerated fish Ringer solution. Square wave stimuli of 330 mA were delivered via parallel platinum electrodes to each muscle preparation, while held at constant length, to generate a series of twitches. Stimulus amplitude (V), pulse width (pulse duration; 2.1 to 2.4 ms) and muscle length were adjusted to determine the stimulation parameters and muscle length corresponding to maximal isometric twitch force. The muscle length that yielded maximal twitch force was measured to the nearest 0.1 mm using an eyepiece graticule fitted to a dissecting microscope. An isometric tetanic force response was then elicited by subjecting the muscle preparation to a 300 ms train of stimulation, using the stimulation amplitude, pulse width and muscle length found to generate maximum twitch force. Time to half peak tetanic force and time from last stimulus to half tetanic force relaxation were measured to the nearest 0.1 ms. A rest period of 5 min was allowed between each tetanic response. Stimulation frequency was then altered (180 to 260 Hz) to determine maximal tetanic force. Rates of force production (peak tetanic force/2 × time to half peak tetanus) and muscle relaxation (peak tetanic force/2 × time from last stimulus to half relaxation) were calculated for the maximal tetanic response. After a further 5 min rest, fatigue resistance was determined by subjecting the muscle preparation to a series of 20 tetani, each of 300 ms stimulation duration, at a rate of one tetanus per second. A further isometric tetanus response was elicited 5 min after the fatigue run. On average, the maximal tetanic force produced by muscle preparations had recovered to 91.5 ± 8.6% (mean ± s.d.) of their pre-fatigue peak by 5 min after the fatigue run.

At the end of the muscle mechanics experiments, bone and connective tissue were removed and each muscle preparation was blotted on absorbent paper to remove excess Ringer solution. Wet muscle mass was determined to the nearest 0.1 mg using an electronic balance. We calculated mean muscle cross-sectional area from muscle length and mass assuming a density of 1060 kg m^−3^. Maximum isometric muscle stress (kN m^−2^) was calculated for each tetanic response as the maximum force within that response divided by mean cross-sectional area.

### 2.5. Behavioural measurements

Fish were introduced to circular, black plastic experimental arenas (diameter 0.9 m, water depth 0.12 m) in groups of four; because sticklebacks are social fish they are most likely to behave naturally in a social context. The water in the arena was aged tap water conditioned to remove additives and at a temperature of 18 ± 1°C. The fish were allowed to settle for 5 min, following which they were filmed for a further 5 min at 1080p HD and 25 frames per second with a digital camera (Lumix FT-4, Panasonic, Japan) positioned above the arena. Following this, the film was converted to .avi format using VirtualDub and the resulting film was tracked using IDTracker [40]. From this, we extracted the mean voluntary speed of each fish and took an average across all fish in each group. Similarly, we measured the inter-individual distances between all fish (all-neighbour distance) and took an average for the group across all time steps. The movement path of animals is intrinsically linked to movement speed [41]. Animals rarely move at maximal speed, but choose a sub-maximal voluntary speed that is linked to environmental contexts [41]. Animals in unfamiliar environments often alter their speed as well as the cohesion of the group in social contexts [42]. Hence, both voluntary speed and group cohesion reflect environmentally induced behavioural changes. We conducted six trials for each of two salinities (0 ppt and 20 ppt) for each of the three environments, making 36 trials in total; we did not re-use individuals for different trials and used a total of 144 fish. Trial order was randomized within salinities.

### 2.6. Statistical analyses

We used discriminant function analysis on Landmark data to compare fish morphology between habitats. The first two discriminant functions explained over 70% of the variance in the data (DF1: eigenvalue = 5.18, %variance = 40.7; DF2: eigenvalue = 3.96, %variance = 31.1) and we used these to compare fish from different habitats (see below).

We analysed all data with Bayesian generalized models or mixed models with Monte Carlo Markov Chain estimation in the package MCMCglmm, R v. 2.22 [43]. For mixed effect models, we implemented 60 000 iterations with a burnin of 10 000, which minimized autocorrelation between posterior samples (assessed from diagnostic plots [43]). We used priors from an inverse Wishart distribution [43]. In cases where the lower Bayesian 95% confidence intervals for the random factor were close to zero, we re-ran the model without the random factor and used the deviance information criterion to determine whether the random factor made a significant contribution to the model fit [44]. Significance was based on a pMCMC value of less than 0.05, and we assessed significant differences between levels of the same factor by comparing the Bayesian 95% confidence intervals of the posterior means.

We compared the two dominant discriminant functions from the Landmark analysis, as well as standard length of fish between habitats (ditch, saltmarsh, river), with habitat as fixed factor and sites within habitats as random factor. We analysed standard length as a dependent variable to determine whether fish length differed between habitats as salinity can affect growth rates and size (see Introduction). *U*_crit_ was analysed with habitat and salinity (fresh and saline water) as fixed factors and site as random factor. Mean voluntary swimming speed and all-neighbour distances in the experimental shoals were analysed with habitat and salinity as fixed factors. For analyses of *U*_crit_, behaviour and discriminant functions, we used fish standard length as covariate. However, to facilitate visual comparisons, we graphed *U*_crit_ and behavioural responses in units of body lengths (BL), and discriminant functions from the morphological analysis as length-corrected residuals.

We compared muscle stress, activation and relaxation rates between fish from the saltmarsh (site 1) and ditch (site 1) with a generalized linear model. To analyse fatigue resistance, we compared stress at the 5, 10, 15 and 20th tetanus as percentage of maximum stress for each muscle preparation between habitats, with habitat as fixed factor and fish id as random factor.

## 3. Results

### 3.1. Morphology

Morphology of fish differed significantly between habitats and most of the variation was captured by the first two discriminant functions based on the Landmark analysis (figure 1*a*,*b*). Negative scores of the first discriminant function (DF1) indicate reduced eye orbit and a small (anterioposteriorally and dorsoventrally compressed) head, and anterioposterior shortening of the posterior body. By contrast, positive scores indicate a relatively enlarged orbit and head, dorsoventral compression of the body and a reduced caudal peduncle (figure 1*b*).

DF1 values for fish from different sites within habitats were similar, that is ‘site’ made a minor contribution to the model only (DIC with site as random factor = 337.7, without = 342.2), but DF1 was significantly different in fish from the saltmarsh habitat compared with river and ditch habitats (*p* < 0.001; figure 1*c*). Saltmarsh fish had strongly positive size-corrected values for DF1 (large head, thin body, small caudal peduncle), while fish from the ditch and river habitats had negative values.

Negative values of the second discriminant function (DF2) indicate an anterioposteriorally compressed head and a relatively deep body at the midsection that is shortened posteriorly with pronounced tapering towards the caudal peduncle and a reduced caudal peduncle. Positive values indicate a long (anterioposteriorly) head, with a dorsoventrally compressed body at the midsection but a distinct peduncle (figure 1*b*).

Site had a significant effect on DF2 (DIC = 398.0 with ‘site’, DIC = 458.4 without ‘site’), and the two river sites were significantly different from each other and from the other habitats (*p* < 0.0001, figure 1*d*). Fish from the river 1 site had strongly negative size-corrected values for DF2 and those from river 2 had positive size-corrected values, while all other fish had values around zero (figure 1*b*–*d*).

Standard length differed significantly between sites (*p* < 0.001), and fish from the river habitat were smallest, followed by ditch and saltmarsh fish (figure 1*e*); sites within habitats were not different from each other (DIC = 670.3 versus 670.1).

### 3.2. Swimming performance

**Figure 2.**
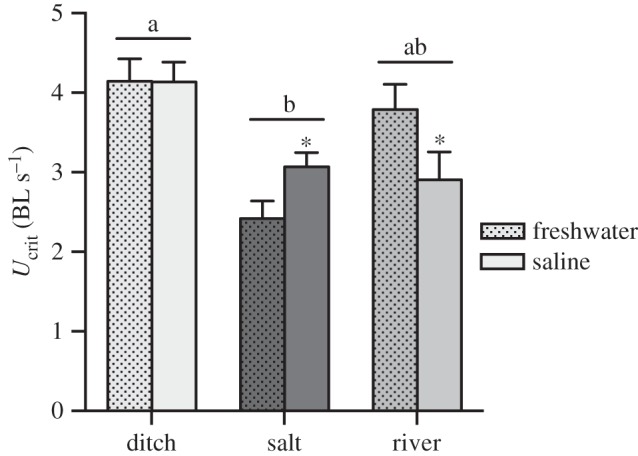
Critical sustained swimming performance (*U*_crit_) of stickleback from different habitats. There were no differences between sites within ditch, saltmarsh (salt) and river environments so that the combined data from both sites within habitats are shown (*n* = 24 fish per habitat and salinity treatment). There was a significant interaction between habitat and salinity, and the fish from the saltmarsh habitat had greater *U*_crit_ in saline water compared with those from freshwater, and the reverse was the case for fish from the river habitat. There was no effect of salinity on swimming performance of ditch fish. Fish from the ditch habitat had significantly greater *U*_crit_ than saltmarsh fish, but neither was different from the river habitat. Means ± s.e. are shown. Horizontal bars with different letters indicate differences between groups of bars and asterisks indicate differences between bars within groups; BL = body lengths.

Swimming performance of fish did not differ between different sites within habitats (DIC with ‘site’ = −524.9, without ‘site’ = −526.8), but there was a significant interaction between habitat and salinity exposure (*p* < 0.01; figure 2). Fish from the saltmarsh had higher *U*_crit_ in saline water while the opposite was the case for river fish and there was no effect of salinity on *U*_crit_ of fish from ditch habitats (figure 2).

### 3.3. Muscle mechanics

We chose to compare the muscle mechanics between fish from saltmarsh (site 1) and ditch (site 1) habitats, because there was no difference between sites within these habitats, and fish from these two sites had the most pronounced differences in morphology (DF1) and swimming performance (figures 1 and 2).

**Figure 3.**
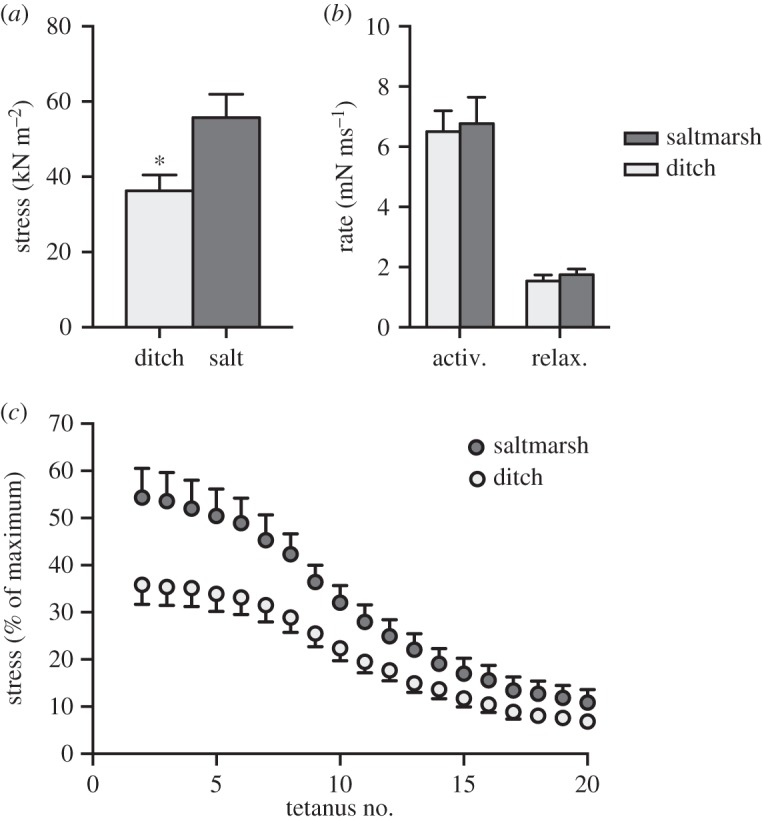
Mechanics of isolated muscle of fish from the ditch and the saltmarsh habitats. Muscle of fish from the ditch habitat produced significantly less stress (force/cross-sectional area) than muscle of saltmarsh fish (indicated by asterisk; (*a*), but there were no differences in the activation and relaxation rates of muscle between the different habitats (*b*). Muscle stress produced relative to maximum for each muscle preparation was greater and declined less rapidly in saltmarsh fish (*c*). Means ± s.e. are shown, *n* = 8 fish per habitat.

**Figure 4.**
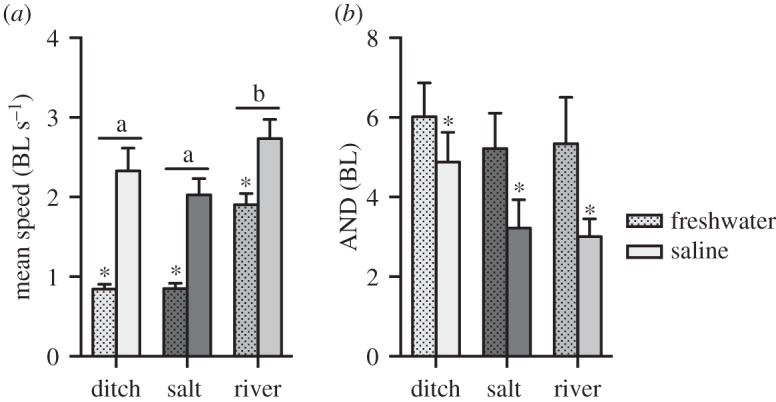
Behaviour of fish shoals from the different habitats. Mean voluntary swimming speed was lower in freshwater compared with saline water, and fish from the river habitat had the highest voluntary swimming speed (*a*). Mean distance between neighbours (all-neighbour distance, AND) was greater in freshwater, but there was no effect of habitat (*b*). Means ± s.e. are shown, *n* = 6 shoals of four fish each per treatment. Horizontal bars with different letters indicate differences between groups of bars and asterisks indicate differences between bars within groups; BL = body lengths.

Muscle stress (i.e. force per unit area) was significantly higher in fish from the saltmarsh habitat compared with those from the ditch (*p* < 0.03; figure 3), but there were no differences in activation (*p* = 0.81) or relaxation (*p* = 0.47) rates between fish from the different habitats (figure 3*b*). Muscle stress decreased with increasing number of tetani and there was a significant interaction between habitat and number of tetani (*p* < 0.0001; figure 4*c*). Saltmarsh fish produced greater stress than ditch fish up to the 10th tetanus, but fish from both habitats produced similar stress by the 20th tetanus (figure 4*c*).

### 3.4. Behaviour

Mean voluntary swimming speed was lower in freshwater compared with saline water (*p* < 0.01) for fish from all habitats, and fish from the river habitat had the highest voluntary swimming speed (*p* < 0.01; figure 4*a*); there was no interaction (*p* = 0.07). Mean distance of the local fish to its neighbours was greater in freshwater (*p* < 0.001; figure 4*b*), but there was no effect of habitat (main effect and interaction *p* > 0.1).

## 4. Discussion

We have shown that there are significant differences in the morphology, physiology and behaviour between fish from different habitats. Habitat-specific morphologies were associated with trade-offs in muscle function and swimming performance. The different habitats were not isolated from each other and fish could move between habitats [45], which indicates that gene flow did not prevent functional stratifications within populations along environmental gradients [46]. The ditch habitats were man-made in the seventeenth century to drain the wetlands [36], which demonstrates that human modification of the environment can elicit functional differences in resident animal populations. The saltmarsh habitat dries sporadically so that fish recolonize this habitat relatively frequently from other habitats. It is unlikely, therefore, that the distinct phenotype of the saltmarsh environment arose solely as a result of local adaptation [2]. It is possible that environmental conditions experienced during the development of individuals and by their immediate ancestors determined phenotypes via epigenetic mechanisms [8,47]. Similarly, stickleback in the St. Laurence estuary showed pronounced differences in response to different salinities in their environment, but there was little evidence that these differences were caused by genetic adaptation [35]. By contrast, genetic differentiation was relatively high in populations of marine stickleback from the Baltic and North Seas. Differentiation was particularly apparent in non-neutral markers, which indicates the occurrence of local adaptation to different salinities in the environment despite gene flow between populations [30,48]. Phenotypic differences between stickleback from different habitats were also associated with genetic stratification in the Misty Lake system in Canada, where fish from the inlet of the lake differed from those inhabiting the lake itself [49]. In the lake environment, migrants from the inlet performed worse than lake fish and phenotypic differences were accompanied by genetic divergence [49,50]. Selection against migrants may have resulted in the observed genetic and phenotypic diversification between habitats [51].

Differences in the genetic structure between local populations [2] may be related to the stability of the environmental signal of the habitat [52,53]. Ephemeral environments that require recurrent colonization like our saltmarsh sites or environments that change across few generations may favour developmental modifiers that match phenotypes to prevailing conditions rather than genetic differences in coding genes resulting from selection [54]. Epigenetic modifications of phenotypes can lead to genetic diversification via genetic assimilation [55–57], and it would be worthwhile to determine experimentally whether or not this mechanism occurs in local adaptation to better understand the process and pace of phenotypic evolution.

The fish sampled in this study were sub-adults and were substantially smaller than those sampled in 2005 and reported in Webster *et al.* [36]. Given the high likelihood of an allometric relationship between body size and morphology, and the lack of overlap in body size between the 2005 and 2015 samples, we opted not to perform a quantitative morphological comparison. Nevertheless, it is informative to draw qualitative comparisons between the two samples. In both the 2005 and 2015 samples, fish from the ditch tended to have deeper bodies and an anterioposteriorally compressed head (prior to size correction, but absent after, described by DF2 in this study). Ditch fish sampled in 2005 had a substantially smaller relative orbit size relative to other fish. This was not so apparent in the 2015 sample, although smaller orbit size in both ditch and river fish relative to fish from the saltmarsh was described by DF1 in this study. Fish from river habitats that were exposed to high predation pressure did not have consistently deeper bodies, which may have conferred an advantage by reducing predation success of gape-limited predators [36]. The difference between the two river sites in 2015 indicates that adaptive responses to predation are not entirely responsible for morphological diversification. Salinity itself can affect morphology and, similar to our results, high salinities have been observed previously to result in slender body shape of stickleback [11].

We have shown that morphological differences were accompanied by differences in locomotor behaviour. We reject our hypothesis that deeper body shape leads to reduced locomotor performance, because fish from the ditch habitat had relatively deeper bodies and also had the highest *U*_crit_. We also reject the hypothesis that fish with deeper bodies have higher performing muscles; ditch fish had muscle that produced less stress than fish from the saltmarsh, which had more slender bodies but also lower *U*_crit_. It is curious that saltmarsh fish with the higher quality muscle also had lower swimming performance relative to ditch fish. The most likely explanation is that the deeper bodies of the ditch fish supported greater quantities of muscle, which compensated for its lower quality. Also, the larger head and smaller caudal peduncle of saltmarsh fish may have resulted in lower swimming performance by increasing drag and reducing thrust, respectively. Stickleback are primarily labriform swimmers that use their pectoral fins for propulsion [16]. However, at higher swimming speeds, caudal fin propulsion becomes increasingly important in labriform swimmers [58,59]. Rates of muscle activation and relaxation can influence muscle power output and therefore locomotor performance [27,60], but neither explained differences between habitats in our stickleback.

We accept the hypothesis that fish specialize in their habitat and fish from the saltmarsh habitat had greater *U*_crit_ in saline water than in fresh water and river fish showed the reverse pattern. Stickleback are a euryhaline species that is distributed across freshwater and marine environments [5]. However, teleost fish, including stickleback, regulate their internal ion concentration so that habitats with different salinities would pose different challenges [28,29]. Active ion exchange occurs mainly in the fish gill, which maintains stability of the internal environment in the face of external osmotic and ionic gradients [61]. Osmoregulatory challenges occur by exposure to both higher and lower salinities than the long-term acclimation conditions. Osmotic challenge, however, does not elicit consistent metabolic responses resulting from increased active ion transport [62,63]. Additionally, the absolute ATP invested into osmoregulation is relatively low [61,62], so that osmoregulation is unlikely to cause an allocation trade-off with locomotion [64] that could explain differences in locomotor performance between populations. However, ATP supply in saline water may be impaired by decreased blood oxygen transport as a result of increased blood volume and reduced haematocrit [65], which may constrain locomotor performance.

Acute exposure to salinity resulted in increased voluntary swimming speeds in fish shoals from all habitats. It may be that fish exposed acutely to more challenging environments increase exploration rates to locate more favourable conditions [66,67]. Increased voluntary speed was associated with decreased distance between members of the shoal. Fish may form more cohesive shoals in more stressful environments [42] and there may be a trade-off with foraging during which shoal become less cohesive [68]. The different swimming speeds of fish from different habitats could also constrain fish from different habitats moving within the same shoal. If that were the case, habitat-specific morphological and physiological differences could lead to behavioural segregation, and this would be an interesting area for future research.

The pronounced morphological and functional differences of fish between different habitats indicate a high degree of plasticity that could increase the resilience of the species to environmental change [8,69]. Morphological differences are fixed within individuals, but responses to salinity can acclimate reversibly within adult organisms [28]. Hence, in complex environments such as our study site, it is likely that phenotypes are determined by responses at different temporal scales, from genetic adaptation to reversible acclimation. The importance of distinguishing between these processes is that the lag between environmental change and phenotypic response differs. Developmental and reversible acclimation act within one or two generations, while adaptation as a result of differential selection would be slower. An important future direction would be to determine the relative contribution of each of these processes, by conducting transplant or common garden experiments [50], for example, because this would show how fast populations can respond to change, especially in human-modified environments.

## Data accessibility

The data accompanying this manuscript have been deposited in Dryad at http://dx.doi.org/10.5061/dryad.kj7p2.
